# Photographic Illustrations of Skin Diseases

**Published:** 1888-03

**Authors:** 


**Photographic Illustrations of Skin Diseases.** Forty-eight quarto plates (seven-six photographic cases from life), accurately colored by hand. An Atlas and Text-Book, 208 pages. By DR. GEO. H. FOX, Professor Diseases of the Skin, College of Physicians aud Surgeons, New York. In twelve numbers, $2.00 each. Complete in one volume. Half roan, $26.75. E. B. Treat, publisher, 771 Broadway, New York.

The first edition of this admirable work, issued eight years ago, was received by the profession and medical press with the highest encomiums. Its value and merits were not only appreciated at home, but also to such an extent abroad that an edition in French was issued by J. B. Bailliere & Son, of Paris, and an edition in German, arranged for by Messrs. F. C. W. Vogel & & Co., of Leipsic. We are heartily glad to welcome the appearance of a second edition, which is not only revised, but made doubly valuable by the substitution of perfect plates for those few which, in the first edition, were open to criticism; the addition of a large number of new ones, and a notable enlargement of the text, so that this edition is an atlas and text-book combined, and covers the whole subject from Acne to Zoster. We cannot too highly recommend this beautiful work.

